# Sexual Dimorphism in Osteoclasts

**DOI:** 10.3390/cells9092086

**Published:** 2020-09-12

**Authors:** Joseph Lorenzo

**Affiliations:** UConn Health, 263 Farmington Avenue, Farmington, CT 06030, USA; jlorenzo@uchc.edu; Tel.: +1-860-679-8199

**Keywords:** osteoclasts, sexual dimorphism, sex steroids, genetics, inflammation

## Abstract

Osteoclasts are the principal mediators of bone resorption. They form through the fusion of mononuclear precursor cells under the principal influence of the cytokines macrophage colony stimulating factor (M-CSF, aka CSF-1) and receptor activator of NF-κB ligand (RANKL, aka TNFSF11). Sexual dimorphism in the development of the skeleton and in the incidence of skeletal diseases is well described. In general, females, at any given age, have a lower bone mass than males. The reasons for the differences in the bone mass of the skeleton between women and men at various ages, and the incidence of certain metabolic bone diseases, are multitude, and include the actions of sex steroids, genetics, age, environment and behavior. All of these influence the rate that osteoclasts form, resorb and die, and frequently produce different effects in females and males. Hence, a variety of factors are responsible for the sexual dimorphism of the skeleton and the activity of osteoclasts in bone. This review will provide an overview of what is currently known about these factors and their effects on osteoclasts.

## 1. Introduction

Osteoclasts are the principal mediators of bone resorption (the process by which bone is removed) [1]. They form predominately under the influence of two cytokines, macrophage colony stimulating factor (M-CSF, aka CSF-1) and receptor activator of NF-κB ligand (RANKL, aka TNFSF11) [2]. Osteoclasts are multinucleated giant cells, which derive from a hematopoietic myeloid-lineage precursor cell that can also differentiate into macrophages and dendritic cells [3]. As a result of their heritage, osteoclasts share a number of characteristics with other innate immune cells. These include the ability to present antigens to T-lymphocytes, the expression of pattern recognition receptors (PRR), like the toll-like receptors (TLR) and the production of proinflammatory cytokines [4]. As the principal mediator of bone resorption, osteoclasts are involved in the development of a number of metabolic bone diseases including osteoporosis and Paget’s disease of bone [5].

**Figure 1:**
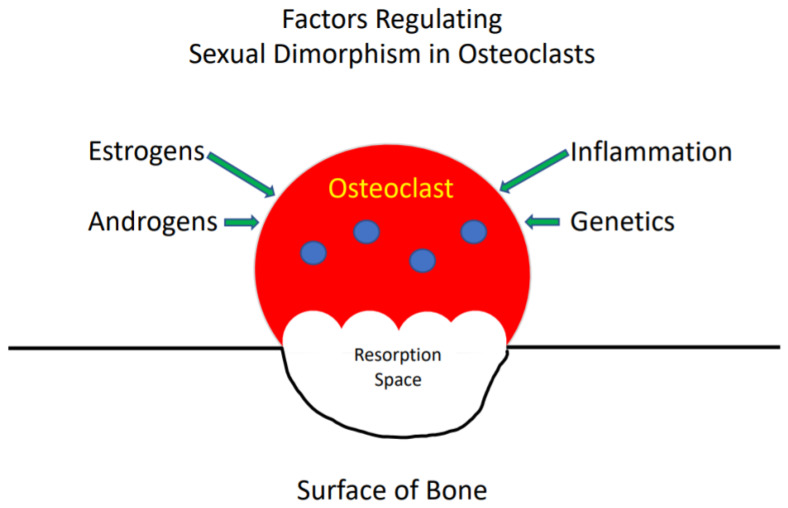
The reasons for the differences between women and men in the bone mass of the skeleton at various ages.

Sexual dimorphism in the development of the skeleton and in the incidence of skeletal diseases is well described [5]. In general, females, at any given age, have a lower bone mass than males [5]. In addition, women predominate in the incidence of osteoporosis while men more frequently develop Paget’s disease of bone. The organization of bone into a functional skeleton, which provides organisms with structural integrity, is the net result of the activity of osteoclasts, which resorb bone, osteoblasts, which form bone and osteocytes, which coordinate the activities of the other two cell types [6]. The reasons for the differences between women and men in the bone mass of the skeleton at various ages and the incidence of certain metabolic bone diseases are multiple and include the actions of sex steroids (estrogens and androgens), genetics and inflammation (Figure 1) [7]. All of these influence the rate that osteoclasts form, resorb and die, and frequently produce different effects in females and males. Hence, a variety of factors are responsible for the sexual dimorphism of the skeleton and the activity of osteoclasts in bone. This review will provide an overview of what is currently known about these factors and their effects on osteoclasts.

## 2. Sexual Dimorphism in the Innate Immune System

Any discussion of the differences between female and male osteoclasts needs to begin with a general overview of the sexual dimorphism of innate immune cells, which share a common origin with osteoclast [7]. Toll-like receptor 7 (TLR7) is encoded on the X chromosome and may escape X-inactivation in certain cell types. For this reason, its levels can be higher in female cells relative to male cells [8]. In contrast, TLR9 responses do not seem to vary between the sexes [9]. TLR signaling pathways in response to stimuli also often demonstrate sexual dimorphism, including higher levels in females of myeloid primary response gene 88 (*MYD88*), retinoic acid-inducible gene-I (*RIGI*), interferon beta (*INFB*), Janus kinase 2 (*JAK2*), signal transducer and activator of transcription 3 (*STAT3*), NF-κB, interferon gamma (*INFG*) and tumor necrosis factor alpha (TNF) [10]. Peritoneal macrophages from males express higher levels of TLR4, which is a receptor for some bacterial cell wall lipopolysaccharides (LPS), and generate higher amounts of CXCX10 with LPS stimulation compared to female cells [11]. Female macrophages also have enhanced phagocytosis and antigen presentation capacity to T-lymphocytes for the initiation of the adaptive immune response [11]. These observed in vitro responses have led to the conclusion that female innate immune cells have an enhanced immune response to common stimuli, compared to male cells. 

## 3. Osteoclast Sexual Dimorphism

My laboratory has found that female-derived murine bone marrow osteoclast precursor cell cultures, treated with M-CSF and RANKL, formed significantly more osteoclasts and demonstrated enhanced resorptive activity relative to males [12]. Our original studies used cultures of bone marrow macrophage (BMM), which are a mixed culture [12]. We have seen similar differences between female and male osteoclastogenesis in cultures of murine bone marrow cells that were directly isolated by fluorescent-activated cell sorting (FACS) as CD11b^lo/neg^, CD3^neg^, CD45R^neg^, CD115 (CSF-1Receptor)^pos^ [13] and then immediately cultured with M-CSF and RANKL for 6 days [14]. The latter assay did not pretreat cells with M-CSF or M-CSF + RANKL to enhance commitment to the macrophage/osteoclast lineage, as is done by some investigators. However, our results are not universal, as some publications found that male cells were more osteoclastogenic, while others found no differences between male and female cells. Valerio et al. [15] examined FACS purified osteoclast precursor cells (OCP) defined as murine bone marrow CD11b^lo^ cells that were first primed with M-CSF and RANKL for 48 h and then stimulated with LPS. They found that in this inflammation assay male cells formed more osteoclasts compared to female cells. In contrast, Zarei A, et al. [16] found no differences in osteoclastogenesis between female and male murine BMM cultures that were first pretreated with M-CSF. These discrepancies probably reflect significant differences in the culture assays that were employed or the origins of the cells. Significantly, our results correlate with measurements of the number of osteoclasts in the bones of mice [14]. However, more work is clearly needed to better understand the reasons for the discrepancies between female and male cultures in the various assays.

## 4. Effects of Sex Steroids on Osteoclasts

### Estrogens

Osteoclasts express estrogen receptor alpha (Erα) [17] and its targeted deletion in myeloid cells in mice, which include the osteoclast precursor, results in a phenotype of increased osteoclast number and decreased trabecular bone mass [18]. The deletion of Erα in myeloid cells produced a bone phenotype that mimicked that of ovariectomized mice. Furthermore, ovariectomizing these mice did not further decrease their trabecular bone mass or increase their trabecular osteoclast number, as it did in wild type mice. These results indicate that the loss of trabecular bone mass in mice is mediated by expression of Erα in myeloid cells, including osteoclasts. Unexpectedly, these authors also found that mice with deletion of Erα in myeloid cells lost cortical bone mass with ovariectomy [18]. Hence, it appears that loss of cortical bone mass in mice is not mediated by expression of Erα in osteoclasts. Using a series of genetic substitutions and specific ligands for nuclear Erα, the authors also demonstrated that non-nuclear Erα binding in myeloid cells was critical for the protective effects of estrogen on trabecular bone.

Estrogens promote apoptosis and inhibit resorption [19] in osteoclasts through mechanisms that depend on Fas ligand (FasL), Fas receptor [20,21,22] and TGFβ [23,24]. The deletion of ERα in mature osteoclasts caused an increase in FasL expression in mice that had been estrogen withdrawn by ovariectomy [20]. In contrast, the deletion of ERα in all myeloid cells, rather than specifically in osteoclasts, did not induce an increase in FasL with estrogen withdrawal [18]. The reasons for this discrepancy are unknown. The effects of estrogen on mitochondrial oxidative phosphorylation in osteoclasts have also been described [25]. It was demonstrated that osteoclasts with deleted ERa in females, but not males, exhibited trabecular bone loss, which was similar to the osteoporotic bone phenotype of postmenopausal women [18,20]. Further, it was shown that estrogen induced apoptosis and upregulated FasL expression in osteoclasts of the trabecular bones of WT, but not ERα deleted mice [20]. FasL production by osteoblasts in response to estrogen has also been shown to regulate osteoclast apoptosis by a paracrine mechanism [21]. Significantly, the latter authors failed to demonstrate upregulation of FasL in osteoclasts with estrogen withdrawal. Hence, this point remains controversial. 

It was also found that antibody inhibition of TGFβ blocked the ability of ovariectomy and its consequent estrogen withdrawal, to prolong the life span of osteoclasts [23]. These effects appear to require interaction of Erα with the adapter protein, breast cancer anti-estrogen resistance protein 1 (BCAR1) [26] and expression of the tyrosine kinase Lyn in osteoclasts [27]. ERβ is also expressed in osteoblasts, osteocytes and osteoclasts [28]. However, its function in these cells is less well understood. There are also effects of estrogen on osteoclastic bone resorption and trabecular, but not cortical bone mass, which are mediated by changes in the permeability of the gut wall to bacterial products and, in turn, alterations of Th17 cell number in Peyer’s patches and T cell TNF production [29].

## 5. Androgens

Loss of androgens in males leads to a decrease in bone mass and an increase in osteoclasts mediated bone resorption [5]. A direct role of androgens on osteoclasts is controversial. Two manuscript found that androgens directly blocked osteoclastogenesis in cultured bone marrow macrophages (BMMs) or RAW264.7 monocyte-macrophage cells [30,31]. This effect was independent of any action of androgens on stromal or osteoblast-lineage cells. Another study using human CD14^+^ peripheral blood monocytes also found direct and dose dependent effects of androgens on in vitro osteoclast formation [32]. A more recent study found that deletion of the androgen receptor (AR), specifically in osteoclasts, had no effect on in vivo osteoclast surface or bone mass [33]. These investigators also found very low expression of the AR in osteoclasts. A second group conditionally deleted the AR either in mesenchymal or myeloid cells in mice, and found that a high turnover, osteopenic trabecular bone phenotype only occurred in mice when AR was deleted in mesenchymal cells [34]. Mice with deletion of the AR in mesenchymal cells were also resistant to trabecular bone loss after orchiectomy. Curiously, these investigators also found that there was no cortical bone phenotype in either of these models (mesenchymal or myeloid AR deletion), and both models lost equivalent amounts of cortical bone with orchiectomy [34]. Hence, the regulation of cortical bone loss with loss of androgens appears independent of AR expression in mesenchymal or myeloid cells.

## 6. Inflammation

Enhanced osteolysis that is driven by inflammation is characteristic of periodontal disease and inflammatory arthritis [35]. As with overall immune responses [10], the osteolytic response to inflammation has been demonstrated to be sexually dimorphic [35]. In models of periodontal disease using *A. actinomycetemcomitans*-derived LPS to enhance RANKL-induced osteoclastogenesis, it was found that the rate of male osteoclastogenesis was greater than that of females [15]. The genes *Nfatc1* and *Tm7sf4* (encoding dendritic cell-specific transmembrane protein or DCSTAMP) were also more highly expressed in male osteoclasts in this model. Likewise, it was found that in a mouse model of pathologic endodontic bone loss, mice with deletion of mitogen-activated protein kinase (MAPK) phosphatase-1 (MKP-1), had greater bone loss in males than in wild type. However, no differences were seen in the bone phenotype between female MKP-1 deficient and wild type mice. MKP-1 is an important negative regulator of the MAPK pathways of the innate immune system [36]. In contrast to these models of inflammatory osteolysis, we found that in mice in homeostasis bone marrow-derived osteoclastogenesis was greater in cells from female than from males [14]. Hence, the model in which osteoclast sexual dimorphism is examined seems to influence what outcome is observed. It is now clear that there are significant differences between the osteoclasts that derive during homeostasis, and those that develop during inflammation [37,38]. These differences in osteoclast origin may, in turn, affect the conflicting results that has been observed in studies of osteoclast sexual dimorphism in murine models.

## 7. Genetics

It has been demonstrated for over 30 years that female mice have more trabecular osteoclasts and a lower bone mass that male mice [39]. There are a variety of reasons for this difference, including sexual dimorphic effects of genes that are expressed in osteoclasts. Treatment of human female and male peripheral blood monocytes with either estrogen or androgen during their in vitro differentiation into osteoclasts identified a number of sexually dimorphic gene expression patterns [40]. A variety of gene deleted mice have also been shown to have sexually dimorphic bone mass or osteoclast phenotypes. Mice with deletion of lysyl oxidases, which is an enzyme that cross-links collagen, demonstrated enhanced osteoclastogenesis and bone loss in females compared to males [41]. Male mice with deletion of transient receptor potential vanilloid 4 (TRPV4) have decreased osteoclasts in their bones and in bone marrow cell cultures relative to females [42]. Caveolae are a specialized type of lipid rafts and expression of caveolin-1 is upregulated by RANKL in developing osteoclasts [43]. Deletion of caveolin-1 in mice resulted in higher bone volume in females, but not males relative to wild type mice [43]. CD59a regulates the membrane attack complex in mice. Its deletion only in male mice produced a bone phenotype of increased cortical bone volume and reduced bone mineral density. In vitro, bone marrow cells from male CD59a-deleted mice demonstrated increased osteoclastogenesis relative to cells from female mice [44]. Disruption of the alternative NF-κB pathway in mice either by global deletion of NF-κB-inducing kinase (NIK) or the NF-κB subunit RelB produced a phenotype of increased bone mass in females only [16]. This was associated with a more sever defect of osteoclastogenesis in female bone marrow cell cultures. Krox20/EGR2 is a zinc finger transcription factor, involved in hindbrain development. Targeted deletion of Krox20 in osteoclast progenitors produced a phenotype of low bone mass and increased resorption only in females [45]. Rac1-specific guanosine triphosphatase (GTPase)-activating protein Slit-Robo GAP2 (Srgap2) is upregulated by RANKL during osteoclastogenesis. Targeted deletion of Srgap2 in osteoclast precursors produced a female-specific high bone mass phenotype [46]. Protein kinase C delta (PKC-δ) deletion in osteoclasts resulted in a high bone mass phenotype only in male mice and an associated decrease in osteoclastogenesis in cultures of male bone marrow cells [47].

## 8. Summary

Clearly, we have much to learn about the mechanisms that regulate the sexual dimorphic responses of osteoclasts. Studies of this phenomenon are important, because they can provide insight into the pathophysiology of metabolic bone diseases like osteoporosis or the response of individuals to therapeutic intervention. Elucidating these mechanisms may identify gene targets that lead to more effective therapies for metabolic diseases of the skeleton.
